# Corrosive Sublimate in Dysentery

**Published:** 1881-08

**Authors:** 


### Corrosive Sublimate in Dysentery.

This remedy, in half-minim doses of the liquor hydrargyri bichloridi of the British Pharmacopeia, administered hourly, speedily and infallibly cures dysentery (barring the tropical and infantile forms), according to Dr. March in the *London Med. Times and Gazette.*

